# Gain-Assisted Giant Third-Order Nonlinearity of Epsilon-Near-Zero Multilayered Metamaterials

**DOI:** 10.3390/nano12193499

**Published:** 2022-10-06

**Authors:** Wenjuan Shi, Hongjun Liu, Zhaolu Wang

**Affiliations:** 1 State Key Laboratory of Transient Optics and Photonics, Xi’an Institute of Optics and Precision Mechanics, Chinese Academy of Sciences, Xi’an 710119, China; 2 University of Chinese Academy of Sciences, Beijing 100084, China; 3 Collaborative Innovation Center of Extreme Optics, Shanxi University, Taiyuan 030006, China

**Keywords:** epsilon-near-zero (ENZ), nonlocal effective medium theory, multilayered metamaterials, third-order nonlinear, gain materials

## Abstract

We investigate the third-order nonlinear optical properties of epsilon-near-zero (ENZ) Au/dye-doped fused silica multilayered metamaterials in the visible spectral range for TM incident by using nonlocal effective medium theory at different incidence angles. The nonlocal response affects the permittivity of anisotropic metamaterials when the thickness of the layer cannot be much smaller than the incident wavelength. By doping pump dye gain material within the dielectric layer to compensate for the metal loss, the imaginary part of the effective permittivity is reduced to 10^−4^, and the optical nonlinear refractive index and nonlinear absorption coefficient are enhanced. The real and imaginary parts of the permittivity are simultaneously minimized when the central emission wavelength of the gain material is close to the ENZ wavelength, and the nonlinear refraction coefficient reaches the order of 10^−5^ cm^2^/W, which is five orders of magnitude larger than that of the nonlinear response of the metamaterial without the gain medium. Our results demonstrate that a smaller imaginary part of the permittivity can be obtained by doping gain materials within the dielectric layer; it offers the promise of designing metamaterials with large nonlinearity at arbitrary wavelengths.

## 1. Introduction

Recently, epsilon-near-zero (ENZ) materials are attracting great interest due to their optical tunneling effect [1], tailoring of radiation patterns [2], enhanced nonlinearity [3,4,5,6,7,8,9,10,11,12,13,14,15], broadband [16] and high-speed tunable devices [17,18,19,20]. Currently, there are two main mechanisms to realize ENZ with different mechanisms and materials: (1) natural materials near the bulk plasma and phonon resonances; (2) anisotropic metamaterials with alternating stacks of negative permittivity metals, positive permittivity dielectrics [21,22,23,24] and metal pillars embedded in dielectrics [25,26]. The ENZ condition can be achieved at any wavelength of interest by reasonably adjusting the thickness of the metal and dielectric layers and the diameter of the metal pillars in the anisotropic metamaterial. The effective medium theory is valid only when the layer thickness of the anisotropic metamaterial is much smaller than that of the wavelength. However, the layer thickness is too thin; in a few nanometers, it leads to the appearance of unwanted effects in materials, such as: dislocations, island formation and size effects [27]. The effect of spatial dispersion, i.e., nonlocal, becomes increasingly important under the ENZ condition of the anisotropic metamaterial; the appearance of strong nonlocal properties significantly alters the optical properties of the material and can lead to the appearance of additional transverse or longitudinal waves [28,29]. Therefore, it is necessary to design the structure with a more suitable nonlocal effective medium theory [30].

ENZ metamaterials vanish a real part of the permittivity, but metals with a large imaginary part of the permittivity have large optical losses, which seriously affects practical applications and is not conducive to improving the nonlinear response. To circumvent these constraints, optical losses can be compensated for by a gain-doped medium with optically pumped dye molecules [31,32,33,34,35,36] or quantum dot [37,38,39,40,41]. Gain materials have little effect on the real part of the permittivity and greatly reduce the imaginary part of the permittivity at the ENZ wavelength. Due to its negative imaginary part and the essentially invariant real part of the effective permittivity, it improves the Kerr-like optical nonlinear response.

In this paper, the linear and nonlinear optical properties of the ENZ anisotropic multilayered metamaterial with alternating Au/dye-doped fused silica at different incidence angles in the visible range for TM light are investigated by considering spatial dispersion using a more comprehensive theoretical approach, i.e., nonlocal effective medium theory. The gain-doped medium reduces the imaginary part of the transverse permittivity to 10^−4^ when the emission wavelength of the gain material is close to the ENZ wavelength. Compared with the metamaterial without gain medium, it has a narrower resonance bandwidth and a 4.92-fold increase in the electric field squared at the ENZ wavelength; the nonlinear coefficient is improved by 5 orders of magnitude.

## 2. Theory and Method

**Figure 1:**
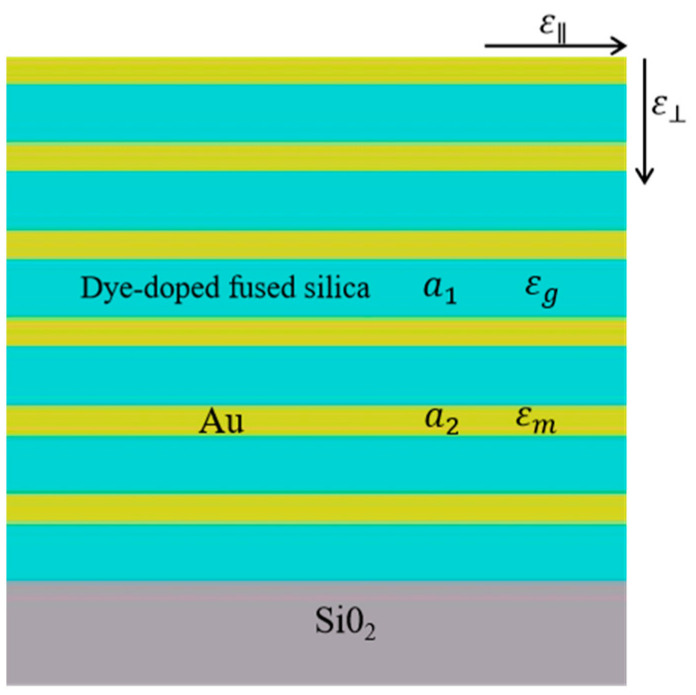
Cross−section of the Au/dye−doped fused silica multilayered metamaterial.

**Figure 2:**
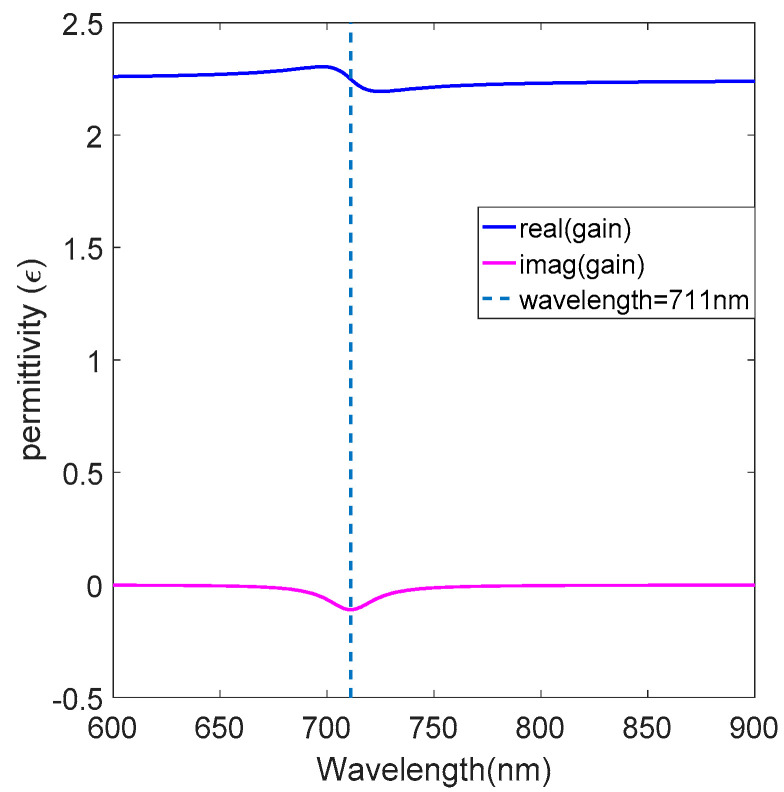
The relative permittivity of the gain−doped medium.

The multilayered metamaterial is composed of alternating Au/dye-doped fused silica with six cycles arranged on the SiO_2_ substrate, as shown in Figure 1. The thickness of gold is 14.9 nm and dye-doped fused silica is 116.2 nm. The optical loss of the metamaterial is compensated for by gain-doped medium with pump dye molecule rhodamine R800. The relative permittivity of this gain-doped medium is a simple Lorentz model [42].

(1) $$\varepsilon_{g}=\varepsilon_{1}\varepsilon_{0}+\frac{\delta_{a}}{\left(\omega^{2}+i\Delta \omega_{a}\omega -\omega_{a}^{2}\right)}\frac{\left(\tau_{21}-\tau_{10}\right)\Gamma_{pump}}{\left[1+\left(\tau_{32}+\tau_{21}+\tau_{10}\right)\Gamma_{pump}\right]}N_{0}$$

where $\Gamma_{pump}$ = $\;\frac{\delta_{abs}I_{pump}}{hf_{30}}$ = 6.5 × 10^9^ s^−1^ is the pumping rate, $\delta_{abs}\;$= 3.14 × 10^−16^ cm^2^ is the absorption cross section, $f_{30}\;$ = 441 THz is the absorption frequency. $\varepsilon_{0}$ is the vacuum permittivity, $\varepsilon_{1}\;$= 2.25 is the dielectric permittivity, $\delta_{a\;}$ is the coupling strength of the polarization density in the emission frequency, $\lambda_{a}=711\;\mathrm{nm}\;,\;\;\omega_{a}\;$= 422 THz is the center emission frequency, $\Delta \omega_{a}$ is the frequency line-width, $\tau_{21}\;$= 500 ps, $\tau_{32}=\tau_{10}=$100 fs, $N_{0}$ is concentration of the R800 and $N_{0}\;$= 1.98 × 10^18^ cm^−3^ corresponds to about 3.3 mM. Figure 2 shows the variation in the relative permittivity for the gain-doped medium with respect to the wavelength, the vertical dashed line is the central emission wavelength and the negative imaginary part of the permittivity clearly shows the loss compensated in the gain-doped medium.

The loss is compensated for when the central emission wavelength of the gain medium is close to the ENZ wavelength of the ENZ multilayered metamaterial. The effective medium theory [43,44] is valid only when the layer thickness is small, and the nonlocal effect is not negligible at larger layer thicknesses and under the ENZ condition, which qualitatively changes the optical properties of the structure. The complex effective permittivity in-plane $\varepsilon_{∥}=\varepsilon_{xx}^{nloc}\;or\;\varepsilon_{yy}^{nloc}$ and out-of-plane $\varepsilon_{⊥}=\varepsilon_{zz}^{nloc}$ is calculated by using the following nonlocal effective medium theory [30].

(2) $$\varepsilon_{xx}^{nloc}=\frac{\varepsilon_{xx}^{loc}-\frac{\alpha }{12}k_{0}^{2}a^{2}}{1-\frac{1}{12}k_{z}^{2}a^{2}}$$

(3) $$\varepsilon_{yy}^{nloc}=\varepsilon_{yy}^{loc}\left(1+\frac{1}{6}k_{x}^{2}a^{2}\right)+\frac{a^{2}}{12k_{0}^{2}}(k_{z}^{4}-k_{x}^{4})-\frac{\alpha }{12}k_{0}^{2}a^{2}$$

(4) $$\varepsilon_{zz}^{nloc}=\frac{\varepsilon_{zz}^{loc}-\frac{\alpha }{12}k_{0}^{2}a^{2}}{1+\frac{\varepsilon_{zz}^{loc}}{\varepsilon_{xy}^{loc}}\left(\frac{\beta }{12}k_{x}^{2}a^{2}-\frac{\gamma }{6}k_{0}^{2}a^{2}\right)}$$

For the metamaterial without gain medium, where α, β, γ,$\;\varepsilon_{xx}^{loc}$, $\varepsilon_{zz}^{loc}$ are:

(5) $$\alpha =\left[f_{m}^{2}\varepsilon_{m}+\left(1-f_{m}^{2}\right)\varepsilon_{d}\right]\left[\left(1-f_{d}^{2}\right)\varepsilon_{m}+f_{d}^{2}\varepsilon_{d}\right]$$

(6) $$\beta =\frac{1}{\varepsilon_{d}\varepsilon_{m}}\left[\left(1-2f_{m}f_{d}\right)\varepsilon_{m}+2f_{m}f_{d}\varepsilon_{d}\right]\left[2f_{m}f_{d}\varepsilon_{m}+\left(1-2f_{m}f_{d}\right)\varepsilon_{d}\right]$$

(7) $$\gamma =\frac{1}{\varepsilon_{d}\varepsilon_{m}}\left[f_{m}^{3}f_{2}\varepsilon_{m}^{3}+f_{m}\left(1-2f_{m}^{2}f_{d}+f_{d}^{3}\right)\varepsilon_{m}^{2}\varepsilon_{d}+f_{d}\left(1-f_{m}f_{d}^{2}+f_{m}^{3}\right)\varepsilon_{m}\varepsilon_{d}^{2}+f_{m}f_{d}^{3}\varepsilon_{d}^{3}\right]$$

(8) $$\varepsilon_{xx}^{loc}=f_{m}\varepsilon_{m}+f_{d}\varepsilon_{d}$$

(9) $$\varepsilon_{zz}^{loc}=\frac{\varepsilon_{d}\varepsilon_{m}}{f_{m}\varepsilon_{m}+f_{d}\varepsilon_{d}}$$

For the metamaterial with gain medium, where α, β, γ,$\;\varepsilon_{xx}^{loc}$, $\varepsilon_{zz}^{loc}$ are:

(10) $$\alpha =\left[f_{m}^{2}\varepsilon_{m}+\left(1-f_{m}^{2}\right)\left(\varepsilon_{1}\varepsilon_{0}+\frac{\delta_{a}}{\left(\omega^{2}+i\Delta \omega_{a}\omega -\omega_{a}^{2}\right)}\frac{\left(\tau_{21}-\tau_{10}\right)\Gamma_{pump}}{\left[1+\left(\tau_{32}+\tau_{21}+\tau_{10}\right)\Gamma_{pump}\right]}N_{0}\;\right)\right]\left[\left(1-f_{d}^{2}\right)\varepsilon_{m}+f_{d}^{2}\left(\varepsilon_{1}\varepsilon_{0}\;+\frac{\delta_{a}}{\left(\omega^{2}+i\Delta \omega_{a}\omega -\omega_{a}^{2}\right)}\frac{\left(\tau_{21}-\tau_{10}\right)\Gamma_{pump}}{\left[1+\left(\tau_{32}+\tau_{21}+\tau_{10}\right)\Gamma_{pump}\right]}N_{0}\;\right)\right]$$

(11) $$\beta =\frac{1}{\varepsilon_{d}\varepsilon_{m}}\left[\;\;\;\;\;\;\left(1-2f_{m}f_{d}\right)\varepsilon_{m}+2f_{m}f_{d}\left(\varepsilon_{1}\varepsilon_{0}+\frac{\delta_{a}}{\left(\omega^{2}+i\Delta \omega_{a}\omega -\omega_{a}^{2}\right)}\frac{\left(\tau_{21}-\tau_{10}\right)\Gamma_{pump}}{\left[1+\left(\tau_{32}+\tau_{21}+\tau_{10}\right)\Gamma_{pump}\right]}N_{0}\;\right)\right]\;\times \;\left[2f_{m}f_{d}\varepsilon_{m}+\left(1-2f_{m}f_{d}\right)\left(\varepsilon_{1}\varepsilon_{0}+\frac{\delta_{a}}{\left(\omega^{2}+i\Delta \omega_{a}\omega -\omega_{a}^{2}\right)}\frac{\left(\tau_{21}-\tau_{10}\right)\Gamma_{pump}}{\left[1+\left(\tau_{32}+\tau_{21}+\tau_{10}\right)\Gamma_{pump}\right]}N_{0}\;\right)\right]$$

(12) $$\begin{array}{cc} \gamma =\frac{1}{\varepsilon_{d}\varepsilon_{m}}[f_{m}^{3}f_{2}\varepsilon_{m}^{3} & +f_{m}\left(1-2f_{m}^{2}f_{d}+f_{d}^{3}\right)\varepsilon_{m}^{2}(\varepsilon_{1}\varepsilon_{0}+\frac{\delta_{a}}{\left(\omega^{2}+i\Delta \omega_{a}\omega -\omega_{a}^{2}\right)}\times \;\frac{\left(\tau_{21}-\tau_{10}\right)\Gamma_{pump}}{\left[1+\left(\tau_{32}+\tau_{21}+\tau_{10}\right)\Gamma_{pump}\right]}N_{0}\;)\\ & +f_{d}\left(1-f_{m}f_{d}^{2}+f_{m}^{3}\right)\varepsilon_{m}{\left(\varepsilon_{1}\varepsilon_{0}+\frac{\delta_{a}}{\left(\omega^{2}+i\Delta \omega_{a}\omega -\omega_{a}^{2}\right)}\times \;\frac{\left(\tau_{21}-\tau_{10}\right)\Gamma_{pump}}{\left[1+\left(\tau_{32}+\tau_{21}+\tau_{10}\right)\Gamma_{pump}\right]}N_{0}\;\right)}^{2}\\ & +f_{m}f_{d}^{3}{\left(\varepsilon_{1}\varepsilon_{0}+\frac{\delta_{a}}{\left(\omega^{2}+i\Delta \omega_{a}\omega -\omega_{a}^{2}\right)}\times \frac{\left(\tau_{21}-\tau_{10}\right)\Gamma_{pump}}{\left[1+\left(\tau_{32}+\tau_{21}+\tau_{10}\right)\Gamma_{pump}\right]}N_{0}\;\right)}^{3}]\; \end{array}$$

(13) $$\varepsilon_{xx}^{loc}=f_{m}\varepsilon_{m}+f_{d}\left(\varepsilon_{1}\varepsilon_{0}+\frac{\delta_{a}}{\left(\omega^{2}+i\Delta \omega_{a}\omega -\omega_{a}^{2}\right)}\frac{\left(\tau_{21}-\tau_{10}\right)\Gamma_{pump}}{\left[1+\left(\tau_{32}+\tau_{21}+\tau_{10}\right)\Gamma_{pump}\right]}N_{0}\;\right)$$

(14) $$\varepsilon_{zz}^{loc}=\frac{\left(\varepsilon_{1}\varepsilon_{0}+\frac{\delta_{a}}{\left(\omega^{2}+i\Delta \omega_{a}\omega -\omega_{a}^{2}\right)}\;\frac{\left(\tau_{21}-\tau_{10}\right)\Gamma_{pump}}{\left[1+\left(\tau_{32}+\tau_{21}+\tau_{10}\right)\Gamma_{pump}\right]}N_{0}\;\right)\varepsilon_{m}}{f_{m}\varepsilon_{m}+f_{d}\left(\varepsilon_{1}\varepsilon_{0}+\frac{\delta_{a}}{\left(\omega^{2}+i\Delta \omega_{a}\omega -\omega_{a}^{2}\right)}\;\frac{\left(\tau_{21}-\tau_{10}\right)\Gamma_{pump}}{\left[1+\left(\tau_{32}+\tau_{21}+\tau_{10}\right)\Gamma_{pump}\right]}N_{0}\;\right)}$$

The permittivity of effective medium theory in-plane and out-of-plane is $\varepsilon_{∥}^{loc}=\varepsilon_{xx}^{loc}=\varepsilon_{yy}^{loc}=\varepsilon_{xy}^{loc}$ and $\varepsilon_{⊥}^{loc}=\varepsilon_{zz}^{loc}$, respectively. $k_{0}\;$=$\;\frac{2\pi }{\lambda }$ is the incident wave vector, $k_{x}\;$=$\;k_{0}\sin\theta_{i}$, $k_{z}\;$=$\;k_{0}\cos\theta_{i},\;\theta_{i}$ is the incident angle, $\varepsilon_{d}$ = 2.25 is the medium relative permittivity, the metal permittivity $\varepsilon_{m}$ can be calculated by the Drude–Lorentz model [45], $f_{m}=\frac{a_{1}}{a}$ is the metal filling ratio, $f_{d}=\frac{a_{2}}{a}$ is the medium filling ratio, $a_{1}$ is the metal thickness, $a_{2}$ is the medium thickness and $a=a_{1}+a_{2}$.

The permittivity of TE and TM waves propagating at the angle of θ in the metamaterial is as follows [25]:

(15) $$\varepsilon_{TE}\left(\theta \right)=\varepsilon_{∥}$$

(16) $$\varepsilon_{TM}\left(\theta \right)=\frac{\varepsilon_{∥}\varepsilon_{⊥}}{\varepsilon_{∥}sin^{2}\left(\theta \right)+\varepsilon_{⊥}cos^{2}\left(\theta \right)}$$

**Figure 3:**
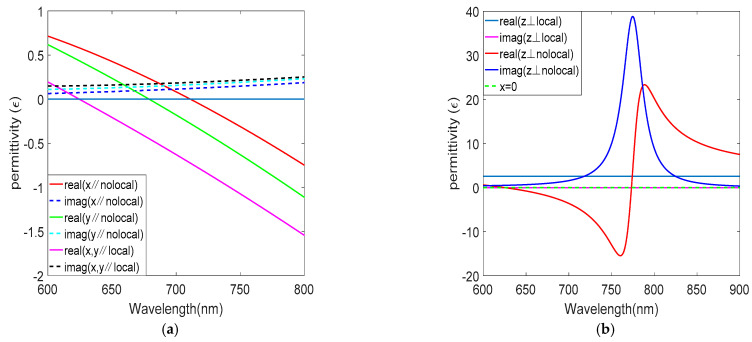
Permittivity without gain medium. (**a**) Permittivity of in−plane local and nonlocal. (**b**) Permittivity of out−of−plane local and nonlocal.

**Figure 4:**
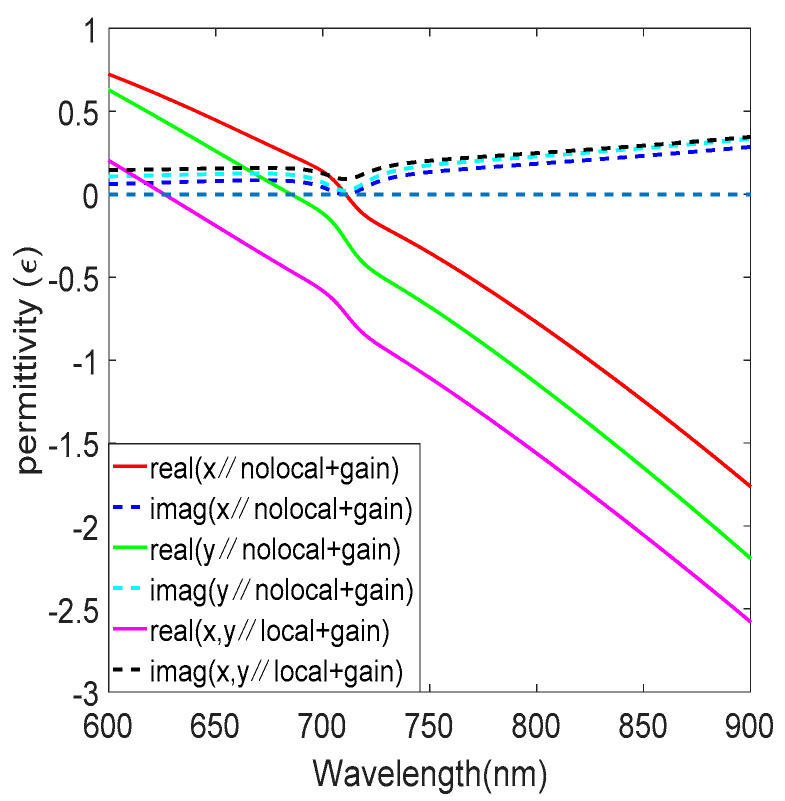
Permittivity of in−plane local and nonlocal with gain medium.

It is worth noting that nonlocal parallel permittivities vary from each other in terms of magnitude, as shown in Figure 3a. This means that nonlocality induces an effective biaxiality of the ENZ multilayered metamaterial, even though the structure is spatially uniform in the XY-plane directions. Compared with the local effective medium theory, the ENZ wavelength occurring within the visible spectral range is red-shifted, and the imaginary part is reduced. The ENZ wavelength in the x-direction is 711 nm, and the imaginary part is 0.1207. The nonlocality has a great influence on the out-of-plane permittivity, as shown in Figure 3b. Since we are particularly interested in how the gain medium affects the nonlinearity, only $\varepsilon_{∥}=\varepsilon_{xx}^{nloc}$ is considered later. Figure 4 shows the relative permittivity versus wavelength after adding the gain medium; the gain medium has little effect on the real part of the permittivity and a great effect on the imaginary part, and the imaginary part of the permittivity is reduced to 5.36 × 10^−4^ at the ENZ wavelength 711 nm.

## 3. Results and Discussions

### 3.1. Linear Response

**Figure 5:**
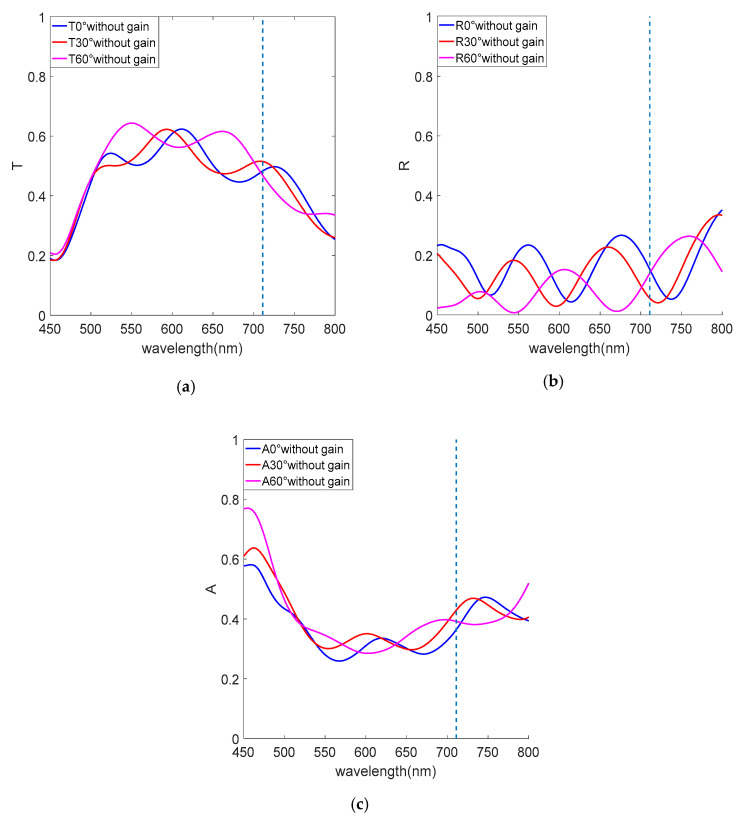
Optical properties without gain medium. (**a**) Transmittance. (**b**) Reflectance. (**c**) Absorption.

The optical properties are affected not only by changing the layer thickness and permittivity, but also by different incidence angles. The characteristics of reflectance R, transmittance T and absorption A of the metamaterial without gain medium for TM wave at different incidence angles are shown in Figure 5, absorption A = 1 – T − R, and the dashed line is the ENZ wavelength 711 nm. The plasmon–polaritonic coupling of the metal–dielectric interface greatly confines the electric field intensity in the dielectric layer [46,47,48,49], leading to multiple distinct resonances of reflectance, transmittance and absorption between wavelengths of 450–800 nm. The metal–dielectric–metal sandwich structure in the multilayered metamaterial is similar to an F-P resonant cavity, which enhances the electric field intensity and, therefore, produces transmittance windows, absorption peaks and reflectance dips near the ENZ wavelength. The resonances of transmittance, reflectance and absorption exhibit a similar blueshift phenomenon with increasing incident angle, and the transmittance of the multilayered metamaterial increases with increasing incident angle, but the reflectance and absorption conversely decrease.

**Figure 6:**
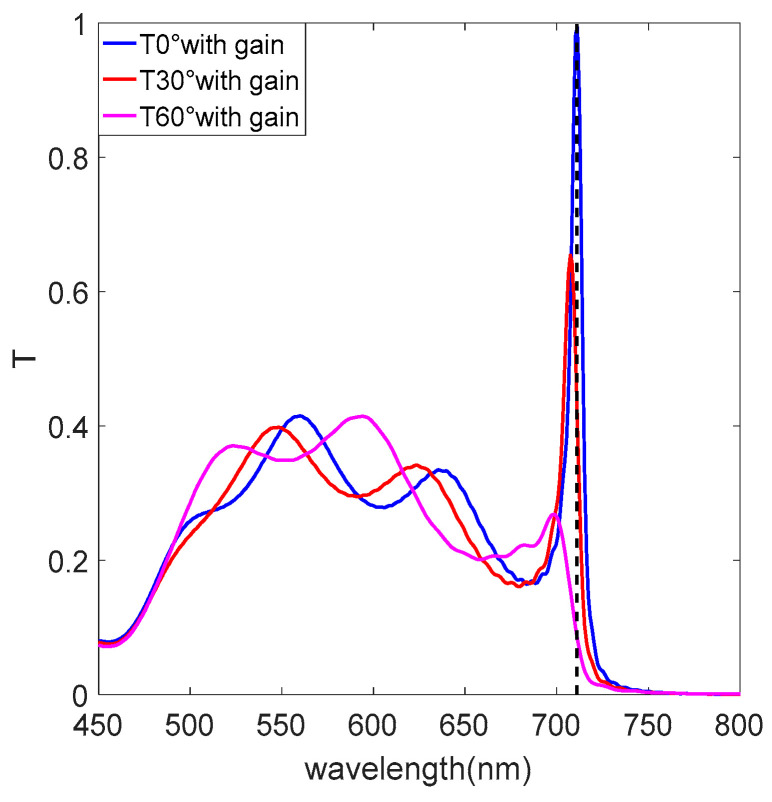
Transmittance characteristic with the gain medium.

**Figure 7:**
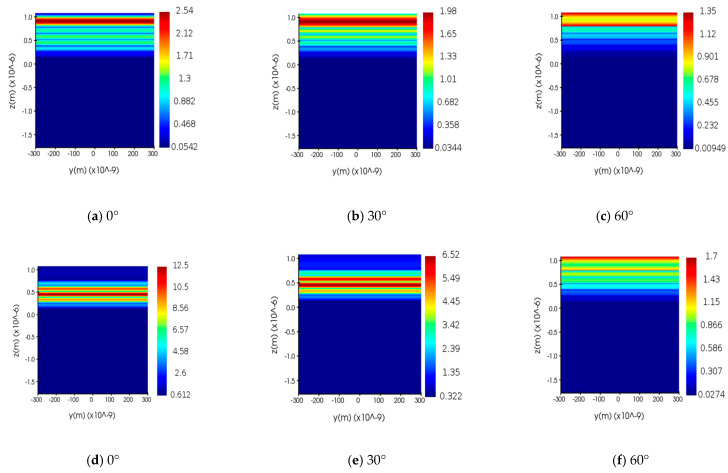
The squared of electric field propagates inside the ENZ multilayered metamaterial for ENZ wavelength at different incidence angles. (**a**–**c**) Without the gain medium. (**d**–**f**) With the gain medium.

In Figure 6, we reveal transmittance characteristics of the structure with gain medium at different incident angles. The transmittance resonance peak at 711 nm in Figure 6 is narrower than that in Figure 5a because of the reduced permittivity after adding the gain medium. The structure also has absorption at 711 nm, but the absorption is smaller than the gain, so there is a narrow-band amplification of the transmittance peak, and transmittance resonance peaks at the remaining wavelengths are similar to Figure 5a, because the permittivity is the same as that of the structure without gain medium. According to the continuity of the electric displacement field for the boundary conditions at the interface for ENZ metamaterials, that is, $\left|E\right|\propto \varepsilon^{-1}\left|E_{0}\right|$, ENZ metamaterials with the gain medium have a strong squared of electric field $({\left|E\right|}^{2}$) enhancement due to the small magnitude of the permittivity (ε), which is 4.92 times larger than in the case without the gain medium at normal incidence. The electric field intensity at the ENZ wavelength also decreases with increasing angle due to the blueshift of the transmittance resonance wavelength and the decrease in the transmittance peak, as shown in Figure 7d–f.

### 3.2. Nonlinear Response

The nonlinear refractive index and nonlinear absorption coefficient of the ENZ multilayered metamaterial were investigated using nonlinear effective medium theory [46]. Here, the effective nonlinear third-order susceptibility $\chi_{eff}^{3}$ of the structure is a weighted average of the constituent materials; since the nonlinear susceptibility of dielectric $\chi_{d}^{\left(3\right)}$ is much smaller than that of the Au $\chi_{m}^{\left(3\right)}$ and can be neglected, only the $\chi_{m}^{\left(3\right)}$ can be considered to the $\chi_{eff}^{3}$ in the ENZ multilayered metamaterial. Au in different material configurations, growth conditions and pulse duration will have different values for the third-order susceptibility, ranging from 10^−14^ to 10^−19^ m^2^V^−2^ [50,51,52,53]. However, we are interested in the effect of the gain medium on the third-order nonlinearity rather than optimizing the optimal third-order nonlinearity. In what follows, we assume that Au is dispersion-less [23] and that the third-order susceptibility of Au is $\chi_{xx}^{\left(3\right)}=\chi_{yy}^{\left(3\right)}=\chi_{zz}^{\left(3\right)}=$ 10^−17^ m^2^V^−2^ [54]. The third-order nonlinear susceptibility of the ENZ multilayered metamaterial parallel to the interface and perpendicular to the interface is as follows [49].

(17) $$\chi_{∥}^{\left(3\right)}=f_{m}\chi_{m}^{\left(3\right)}$$

(18) $$\chi_{⊥}^{\left(3\right)}=f_{m}\chi_{m}^{\left(3\right)}{\left|\frac{\varepsilon_{⊥}^{nloc}}{\varepsilon_{m}}\right|}^{2}{\left(\frac{\varepsilon_{⊥}^{nloc}}{\varepsilon_{m}}\right)}^{2}$$

where $\frac{\varepsilon_{⊥}^{nloc\;}}{\varepsilon_{m}}$ displays the local field enhancement factor in the metallic component.

For the TM wave at all incidence angles, the third-order susceptibility is derived as follows:

(19) $$\varepsilon_{TM}^{NL}\left(\theta \right)\;=\frac{\varepsilon_{∥}^{NL}\varepsilon_{⊥}^{NL}}{\varepsilon_{∥}^{NL}sin^{2}\left(\theta \right)+\varepsilon_{⊥}^{NL}cos^{2}\left(\theta \right)}$$

where $\varepsilon_{∥}^{NL}$ and $\varepsilon_{⊥}^{NL}$ are the in-plane and out-of-plane nonlinear permittivity, respectively [23]:

(20) $$\varepsilon_{∥}^{NL}\;=\varepsilon_{∥}+3\chi_{∥}^{\left(3\right)}\sin^{2}\left(\theta \right){\left|E\right|}^{2}$$

(21) $$\varepsilon_{⊥}^{NL}\;=\varepsilon_{⊥}+3\chi_{⊥}^{\left(3\right)}\sin^{2}\left(\theta \right){\left|E\right|}^{2}$$

By replacing $\varepsilon_{∥}^{NL}$ and $\varepsilon_{⊥}^{NL}$ in Equation (19) with Equations (20–21), the nonlinearity in the denominator is too small compared with linearity, so the effects of the nonlinearity in the denominator and the electric field ${\left|E\right|}^{4}$ can be neglected.

(22) $$\varepsilon_{TM}^{NL}\left(\theta \right)\approx \varepsilon_{TM}\left(\theta \right)+3\varepsilon_{TM}\left(\theta \right)\sin^{2}\left(\theta \right)\left(\frac{\chi_{∥}^{\left(3\right)}}{\varepsilon_{∥}^{nloc}}+\frac{\chi_{⊥}^{\left(3\right)}}{\varepsilon_{⊥}^{nloc}}\right){\left|E\right|}^{2}$$

To make a further approximation, assuming that the nonlinear component is much smaller than the linear component, Snell’s law can be written as $\varepsilon_{TM}^{NL}\left(\theta \right)\sin^{2}\left(\theta \right)$=$\varepsilon_{TM}\left(\theta \right)\sin^{2}\left(\theta \right)$=$\varepsilon_{i}\sin^{2}\left(\theta_{i}\right)$. $\theta_{i}$ is the angle of incidence and $\varepsilon_{i}$ is the permittivity of the air medium, therefore, Equation (22) can be rewritten as:

(23) $$\varepsilon_{TM}^{NL}\left(\theta_{i}\right)\approx \varepsilon_{TM}\left(\theta_{i}\right)+3\varepsilon_{i}\sin^{2}\left(\theta_{i}\right)\left(\frac{\chi_{∥}^{\left(3\right)}}{\varepsilon_{∥}^{nloc}}+\frac{\chi_{⊥}^{\left(3\right)}}{\varepsilon_{⊥}^{nloc}}\right){\left|E\right|}^{2}$$

(24) $$\chi_{TM}^{\left(3\right)}\left(\theta_{i}\right)\approx \varepsilon_{i}\left(\frac{\chi_{∥}^{\left(3\right)}}{\varepsilon_{∥}^{nloc}}+\frac{\chi_{⊥}^{\left(3\right)}}{\varepsilon_{⊥}^{nloc}}\right)$$

The complex nonlinear response is:

(25) n˜2=34cε0Re(n0)n0χeff3

where $\varepsilon_{0}$ is the permittivity in a vacuum, $n_{0}$ is the complex effective linear refractive index, Re(n0) is the real part of the complex effective linear refractive index and c is the speed of light. The nonlinear refraction coefficient and nonlinear absorption coefficient are as follows:

(26) $$n_{2}=Re\left({\tilde{n}}_{2}\right)$$

(27) β=4πλIm(n˜2)

**Figure 8:**
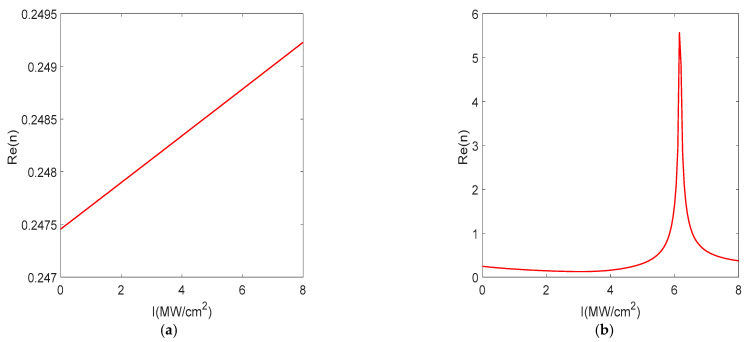
Intensity−dependent refraction index of the multilayered metamaterials at 710.8 nm. (**a**) Without the gain medium. (**b**) With the gain medium.

We investigated the intensity-dependent refraction index *n* of the multilayered metamaterial. The nonlinear refractive index change is greater than the linear refractive index change under ENZ conditions, which violates the nonlinear perturbation theory [3]. Therefore, the conventional intensity-dependent refractive index, $n\left(I\right)=n_{0}+n_{2}I$, is not applicable; we directly use the susceptibility to calculate the intensity-dependent refractive index, i.e., $n\left(I\right)=\sqrt{\varepsilon^{\left(1\right)}+3\chi^{\left(3\right)}{\left|E\right|}^{2}}$. The incident light intensity *I* directly affects the permittivity of the gain medium as in Equation (1). The permittivity imaginary $Im(\varepsilon_{∥})$ is closest to 0 as the incident light intensity increases to 6.15 MW/cm^2^, and the refractive index *Re(n)* = 5.57 is at its maximum at *I* = 6.15 MW/cm^2^, as shown in Figure 8b, but the nonlinear bandwidth becomes narrower than *I* = 6.05 MW/cm^2^. When *I* > 6.15 MW/cm^2^, *Re(n)* decreases due to the increase in $Im(\varepsilon_{∥})$. We use *I* = 6.05 MW/cm^2^ in the following after considering the nonlinear coefficient and the bandwidth size. For metamaterials without the gain medium, *Re*(n) in Figure 8a is much smaller than that in Figure 8b with the gain medium. The above results show that multilayered metamaterials with gain media exhibit an order of magnitude of nonlinear refractive index change under ENZ conditions.

**Figure 9:**
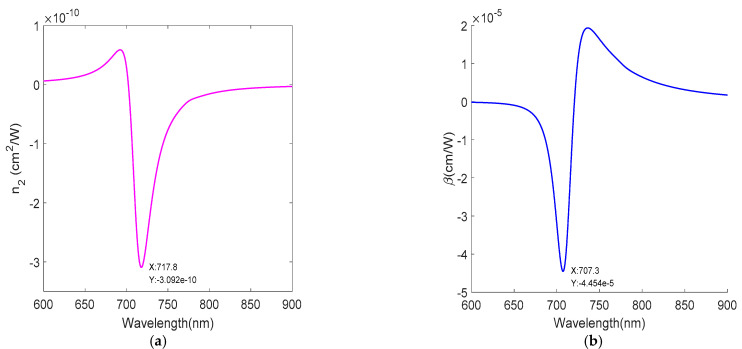
Nonlinear coefficient without the gain medium. (**a**) Nonlinear refraction index $n_{2}$. (**b**) Nonlinear absorption coefficient β.

**Figure 10:**
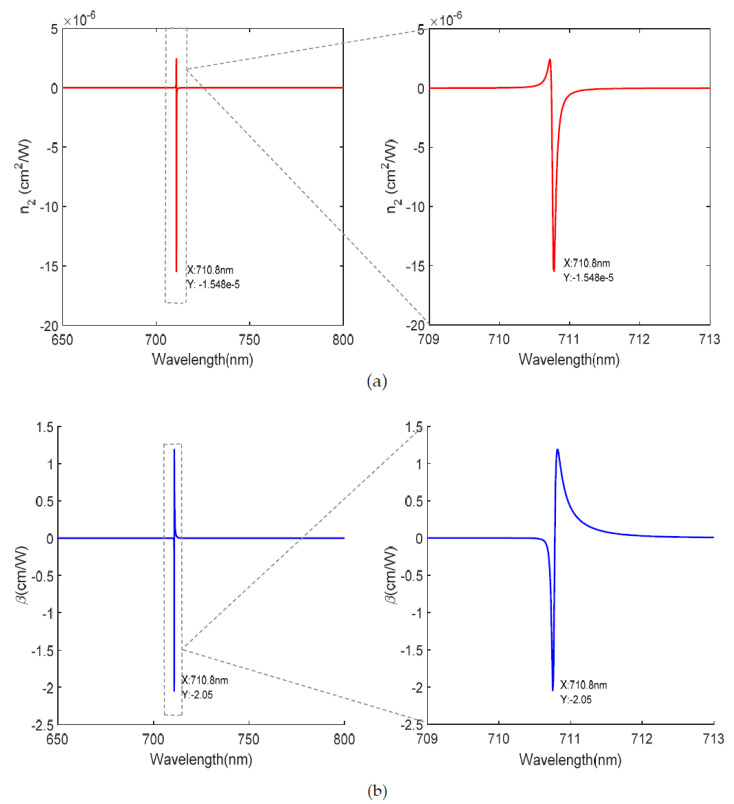
Nonlinear coefficient with the gain medium. (**a**) Nonlinear refraction index $n_{2}$. (**b**) Nonlinear absorption coefficient β.

The metamaterial displays a strong wavelength-dependent nonlinear response; the conversion between self-focusing to self-defocusing and saturated absorption and anti-saturated absorption can be achieved by controlling the wavelength. For the metamaterial without gain medium, the $\left|n_{2}\right|$ is the maximum and reaches the order of 10^−10^ cm^2^/W at the wavelength of 717.8 nm, as shown in Figure 9a. For the metamaterial with gain medium, as shown in Figure 10a, the complex linear refractive index is well reduced and the third-order nonlinear susceptibility is increased due to the reduction in the imaginary part of the dielectric constant at 710.8 nm, thus, the $\left|n_{2}\right|$ is the maximum and reaches the order of 10^−5^ cm^2^/W, which is an increase of 5 orders of magnitude compared with the nonlinear refractive index for normal incidence in Figure 9a. Similarly, for the metamaterial without gain medium, the |β| at 707.3 nm is the maximum and reaches the order of 10^−5^ cm/W, as shown in Figure 9b. For the metamaterial with gain medium, as shown in Figure 10b, the |β| is the maximum at the wavelength of 710.8 nm and reaches the order of 1 cm/W, which is an increase of 5 orders of magnitude in the nonlinear absorption coefficient compared with Figure 9b. Table 1 shows the comparisons of nonlinear coefficients (nonlinear refraction coefficient and nonlinear absorption coefficient) for similar systems, and the results of this article are shown in bold. The nonlinear refraction coefficient and nonlinear absorption coefficient reach the order of 10^−5^ cm^2^/W and 1 cm/W, respectively, which are several orders of magnitude larger than the results from other literature. Therefore, the gain-doped medium improves the nonlinear response, and the dominant mechanism for improving the nonlinearity is the factor 1/Re(n0)n0.

.

**Figure 11:**
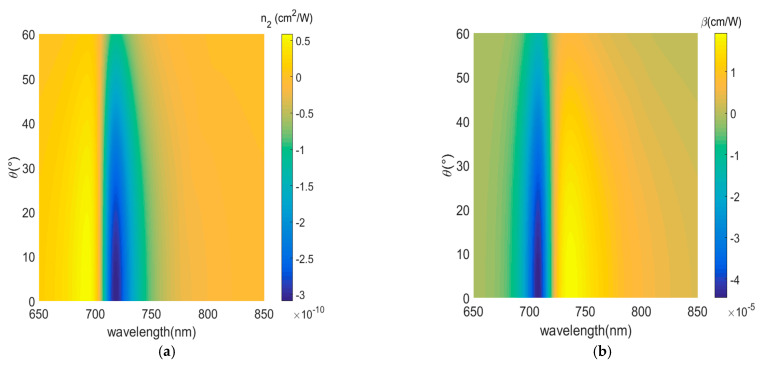
Nonlinear coefficient as a function of incident angle and gain medium concentration (0 mM). (**a**) Nonlinear refraction index $n_{2}.$ (**b**) Nonlinear absorption coefficient β.

**Figure 12:**
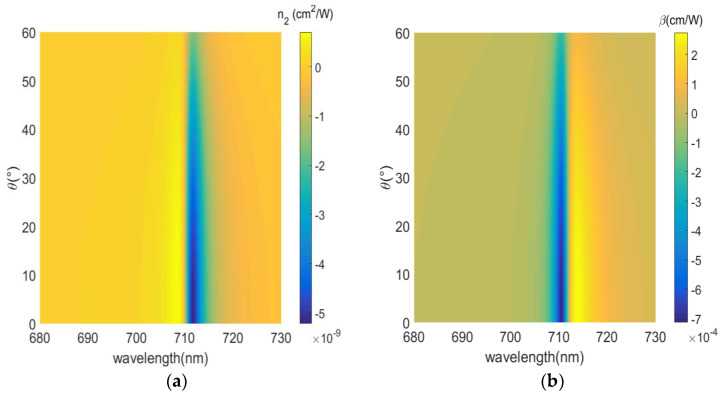
Nonlinear coefficient as a function of incident angle and gain medium concentration (2.5 mM). (**a**) Nonlinear refraction index $n_{2}$. (**b**) Nonlinear absorption coefficient β.

**Figure 13:**
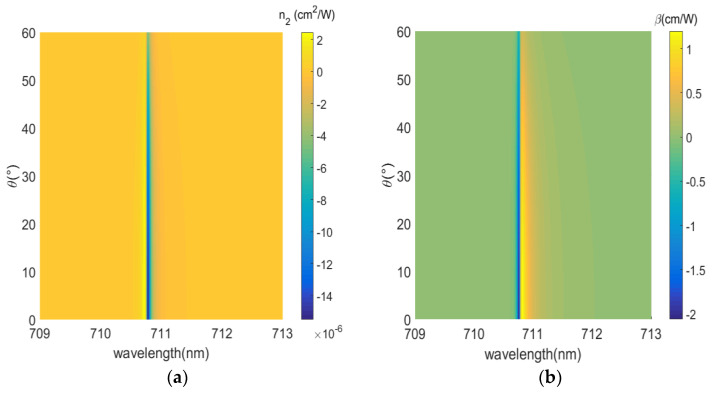
Nonlinear coefficient as a function of incident angle and gain medium concentration (3.3 mM). (**a**) Nonlinear refraction index $n_{2}.$ (**b**) Nonlinear absorption coefficient β.

Finally, we compare nonlinear coefficients of gain media with different incidence angles and different concentrations: 0 mM in Figure 11, 2.5 mM in Figure 12, 3.3 mM in Figure 13. Since the imaginary part of the permittivity increases and the electric field intensity coupled in the medium decreases with the increasing angle, the absolute value of the nonlinear coefficient decreases over the entire wavelength range. We consider concentrations of R800 dye molecules in the dielectric layer from 0 mM to 3.3 mM; on the one hand, the reduction in the imaginary part of the effective permittivity in the plane, $Im\left(\varepsilon_{∥}\right)\;$≈ 0.1207 for 0 mM to $Im\left(\varepsilon_{∥}\right)$ ≈ 10^−4^ for 3.3 mM, i.e., the loss is well compensated. On the other hand the narrower resonance bandwidth leads to an increase in the intensity of the coupled electric field in the dielectric layer with increasing concentration of the gain medium. The nonlinear coefficients, near the ENZ wavelength, are increased by 1 order and 5 orders of magnitude for concentrations of 2.5 mM and 3.3 mM, respectively. Further, a broadband nonlinear response is shown for 0 mM concentration. When the gain medium concentration increases and loss is compensated, the nonlinear displays an angle/spectrum narrowband characteristic. Therefore, the nonlinearity of the ENZ multilayered metamaterial must be improved with an appropriate concentration of the gain medium. Table 2 shows the comparisons of nonlinear coefficients (nonlinear refraction coefficient, nonlinear absorption coefficient) at different doping concentrations.

## 4. Conclusions

In conclusion, the linear and nonlinear properties of the ENZ multilayered metamaterial are investigated based on the nonlocal effective medium theory, and the ENZ wavelength of interest can be adjusted arbitrarily by adjusting the thickness of the dielectric and metal layers. The metal–dielectric–metal in the ENZ multilayered metamaterial is similar to the F-P cavity; the electric field intensity can be coupled in the dielectric layer, but metals have large optical losses, leading to a large imaginary part of the permittivity, which seriously affects practical applications and is not conducive to improving the nonlinear response. Therefore, the doping of R800 dye molecules with a concentration of 3.3 mM in the dielectric layer can be used to reduce the imaginary part of the permittivity in the ENZ multilayered metamaterial to 5.36 × 10^−4^. Compared with the metamaterial without gain medium, the squared electric field is enhanced by 4.92 times, and the nonlinear coefficient is increased by 5 orders of magnitude. Moreover, the conversion of self-focusing and self-defocusing as well as saturable absorption and anti-saturable absorption can be achieved by controlling the wavelength, and the absolute value of the nonlinear coefficient decreases with increasing angle. We believe that our investigation of the gain-doped ENZ multilayered metamaterial can lead to important applications in nonlinear optics and quantum optics.

## Figures and Tables

**Table 1:** Comparisons of the third-order nonlinear optical coefficients of different references.

**References**	Structures	$\mathrm{Wavelength}\;\left(\mathrm{nm}\right)/n_{2}({\mathrm{cm}}^{2}/W)$	Wavelength (nm)/β (cm/W)
**Our work**	Multilayered metamaterial with a gain medium	710.8/10^−5^	710.8/10^0^
[3]	ITO film	1240/10^−10^	1260/10^−6^
[4]	AZO film	1310/10^−13^	1340/10^−8^
[5]	Metasurface	1240/10^−9^	1260/10^−5^
[10]	Metasurface	1250/10^−9^	1300/10^−4^
[23]	Multilayered metamaterial	470/10^−8^	470/10^−3^
[24]	Multilayered metamaterial	500/10^−9^	500/10^−4^

**Table 2:** Comparisons of the third-order nonlinear optical coefficients of different doping concentrations.

Concentrations	$n_{2}({\mathrm{cm}}^{2}/W)$	β (cm/W)
3.3 mM	10^−5^	10^0^
2.5 mM	10^−9^	10^−4^
0 mM	10^−10^	10^−5^

## Data Availability

The data presented in this study supporting the results are available in the main text. Additional data are available on request to the corresponding author.
